# Gut Dysbiosis Is Associated With the Severity of Cryptogenic Stroke and Enhanced Systemic Inflammatory Response

**DOI:** 10.3389/fimmu.2022.836820

**Published:** 2022-05-06

**Authors:** Qianyi Zheng, Yongkang Chen, Yanping Zhai, Lin Meng, Han Liu, Haiyan Tian, Renyi Feng, Jiuqi Wang, Rui Zhang, Kedi Sun, Lina Gao, Yijing Wang, Xuejing Wang, Erxi Wu, Junfang Teng, Xuebing Ding

**Affiliations:** ^1^Department of Neurology, The First Affiliated Hospital of Zhengzhou University, Zhengzhou, China; ^2^Institute of Parkinson and Movement Disorder, Zhengzhou University, Zhengzhou, China; ^3^Neuroscience Institute and Department of Neurosurgery, Baylor Scott & White Health, Temple, TX, United States; ^4^College of Medicine, Texas A&M University, College Station, TX, United States; ^5^Irma Lerma Rangel College of Pharmacy, Texas A&M University, College Station, TX, United States; ^6^LIVESTRONG Cancer Institutes and Department of Oncology, Dell Medical School, University of Texas at Austin, Austin, TX, United States

**Keywords:** gut microbiota, ischemic stroke, cryptogenic stroke, systemic inflammatory response, gut inflammation

## Abstract

Studies implicate that gut dysbiosis is related with many neurological diseases. However, the potential role of gut dysbiosis in cryptogenic stroke (CS) has not been elucidated yet. In this study, a high prevalence of gastrointestinal (GI) dysfunction and gut inflammation with increased intestinal permeability have been found in CS patients compared with normal controls (NCs). The systemic inflammation in CS patients was also identified by measuring the levels of plasma C-reactive protein (CRP), lipopolysaccharide (LPS), LPS-binding protein (LBP), and white blood cells (WBC) count. Using 16S rRNA sequencing, we found increased alpha diversity, accompanied by a higher abundance of Enterobacteriaceae, Streptococcaceae, and Lactobacillaceae at the family level and *Escherichia–Shigella*, *Streptococcus*, *Lactobacillus*, and *Klebsilla* at the genus level in the intestinal microbiota of CS patients compared to NCs. Our results showed that the abundance of *Klebsilla* was positively correlated with the systemic inflammation, the National Institutes of Health Stroke Scale (NIHSS) scores, and the infarct volumes. In conclusion, gut dysbiosis in CS patients was associated with the severity of CS and the systemic inflammation. Maintaining the intestinal homeostasis may be a potential strategy for the treatment of CS.

## Introduction

Despite extensive clinical examination, a considerable proportion of ischemic strokes were classified as having an undetermined cause and were identified as cryptogenic stroke (CS) (1, 2). Compared to strokes of identified cause, CS results in less severe symptoms and lower mortality. It has been reported the proportion of CS in all ischemic strokes ranges from 23% to 40% (1, 3, 4). Despite the high incidence of CS, it has scarcely been studied.

It is increasingly recognized that intestinal pathological changes are correlated to neurological diseases, such as cavernous angioma (CA) (5), Parkinson’s disease (PD) (6), and Alzheimer’s disease (AD) (7). The gastrointestinal (GI) tract is functionally connected with the brain through the gut–brain axis (GBA) (8). Several studies indicated that patients are susceptible to GI complications after stroke, including dysphagia, constipation, and bleeding (9, 10). Besides the disturbance of GI function, alteration of gut microbiota after stroke has also been paid attention to. Disturbances within the GBA (11), especially the gut microbiota dysbiosis, have been reported in patients with ischemic stroke (12). Moreover, alteration of cecal microbiota was presumed to play roles in the onsets and development of ischemic stroke in an animal study (13).

Recently, researchers have found that gut dysbiosis and systemic inflammation may have an intimate connection in animal models (14, 15). Shifts in the makeup of gut microbiota could induce increased intestinal permeability and systemic inflammation (15–17). Inflammation has been reported to play an important role in the pathogenesis of ischemic stroke (18, 19) and also emerging as a predisposing factor for stroke (20). Animal studies showed that systemic inflammation could increase the risk of stroke and are associated with less favorable clinical outcomes (21, 22). However, little attention has been paid to the role of gut microbiota changes and systemic inflammation in the process of ischemic stroke in patients, let alone in CS patients.

In the present study, we sought to investigate whether and how gut dysbiosis and systemic inflammation are developed in CS patients and, if so, to further identify the link with the severity of CS.

## Materials and Methods

### Subjects

CS patients diagnosed and treated in the Department of Neurology at the First Affiliated Hospital of Zhengzhou University from February 2021 to September 2021 were enrolled. Patients aged 18–45 years and with first-ever acute ischemic stroke diagnosed by two neurologists were initially recruited. Patients routinely underwent brain MRI, magnetic resonance angiography (MRA), carotid duplex ultrasonography, 12-lead ECG, and laboratory blood test (i.e., full blood count, white blood cells (WBC) count, clotting, C-reactive protein (CRP), erythrocyte sedimentation rate, liver function, renal function, thyroid function, electrolytes, and lipid profile) after the event. The cause of the stroke was classified according to the modified Trial of Org 10172 in Acute Stroke Treatment (TOAST) criteria (23). We classified patients as cryptogenic if the diagnostic workup included at least brain imaging, ECG, and extracranial imaging and if no clear cause was found. Exclusion criteria were as follows: i) stoke with determined cause, ii) lacunar brain infarction, iii) history of ischemic stroke or transient ischemic attack (TIA), iv) other neurological diseases (such as PD and AD), v) recent (within 3 months) infection, vi) recent use of antibiotics or probiotics, vii) history of GI operation, viii) history of inflammatory bowel disease, and ix) pregnancy. Stroke severity at admission was assessed by a certified neurologist using the National Institutes of Health Stroke Scale (NIHSS) (0–42; the higher the score, the more serious the disease). Gastrointestinal Symptom Rating Scale (GSRS) was used to assess the severity of GI dysfunction.

Normal controls (NCs) were recruited from the Physical Examination Department of the First Affiliated Hospital of Zhengzhou University. They had undergone a series of tests including a physical exam and laboratory testing, such as blood and urine routine, clotting, CRP, erythrocyte sedimentation rate, blood glucose and lipids, thyroid function, liver and kidney function, MRI, and MRA scan. All control subjects were confirmed to be free of neurological and GI disorders, as determined by two attending neurologists and a gastroenterologist. Finally, age, sex, and stroke risk factor frequency-matched subjects were enrolled as controls in this study.

This study was authorized by the Institutional Ethics Committees of The First Affiliated Hospital of Zhengzhou University, and informed consent was obtained from all participants (number: 2021-KY-0387-002).

### Imaging Analysis

The infarct volumes on diffusion-weighted imaging (DWI) were measured by an experienced neurologist unaware of the clinical and laboratory results. The infarct volume was calculated by using the formula 0.5 × a × b × c (where a is the maximal longitudinal diameter, b is the maximal transverse diameter perpendicular to a, and c is the number of 10-mm slices containing infarct) according to the DWI sequences.

### Sample Collection

Plasma samples were obtained on admission within 72 h from each subject *via* venipuncture. Samples were collected in endotoxin-free K2 EDTA 10-ml tubes. Each sample was centrifuged at 2,000×*g* for 10 min, plasma aliquoted, and stored at −80°C. Extreme care was taken to keep all samples sterile and endotoxin/lipopolysaccharide (LPS) free, and all processing was performed using sterile, LPS-free reagents and plastic ware.

Each participant was asked to collect a stool sample of approximately 5 g within 48 h after admission using fecal collection containers. Then containers were transferred on ice and stored at −80°C before processing. Before measuring, stool samples were preliminarily processed to get the supernatant. Specifically, fecal samples were weighed and then homogenized in phosphate-buffered solution (PBS) (pH = 7.4) (tissue weight (g): PBS (ml) volume = 1:9) with a glass homogenizer on ice. To further break down the cells, the suspension was sonicated with an ultrasonic cell disrupter. The homogenates were then centrifuged for 10 min at 5,000×*g* to get the supernatant.

Intestinal biopsy specimens were taken in the colon during a colonoscopy for 25 NCs and 26 CS patients. Samples were then fixed in 4% paraformaldehyde and embedded by paraffin for immunofluorescence experiments or directly stored at −80°C for Western blotting analysis.

### Detection of Biomarkers

After species were collected, the quantitative evaluations of biomarkers in plasma and feces were performed by ELISA tests as per the manufacturer’s instructions: Human Lipopolysaccharide/Endotoxin (LPS/ET) ELISA Kit, Human LPS-binding protein (LBP) ELISA Kit, Human Lactoferrin ELISA Kit, Human Calprotectin ELISA Kit, Human Alpha 1-Antitrypsin ELISA Kit and Human Zonulin ELISA Kit. All operations follow the manufacturer’s protocol.

### Analysis of Gut Microbiota

16S rRNA genes of region 16S V3–V4 were amplified using a specific primer (341F-806R) with the barcode. Sequencing libraries were generated using TruSeq^®^ DNA PCR-Free Sample Preparation Kit (Illumina, San Diego, CA, USA) following the manufacturer’s recommendations, and index codes were added. Paired-end read assembly and quality control were respectively performed by FLASH (V1.2.7) (24) and QIIME (V1.9.1) (25) quality-controlled process. Sequence analyses were performed by Uparse software (Uparse v7.0.1001) (26). Sequences with ≥97% similarity were assigned to the same operational taxonomic units (OTUs). OTU abundance information was normalized using a standard sequence number corresponding to the sample with the least sequences. Alpha diversity indices were calculated with QIIME (Version 1.7.0) and displayed with R software (Version 2.15.3). Beta diversity analysis was QIIME (Version 1.9.1) and displayed with R software (Version 2.15.3).

### Histopathological Study

Colon serial sections (4 μm in thickness) mounted on probe-on slides were deparaffinized in xylene and rehydrated in a series of graded ethanol solutions. Then sections were stained with H&E. For each sample, the microscopic damage score investigated was assessed blindly by two investigators by light microscopy. Colon sections were assessed for quantitative analysis of intestinal inflammation, according to a microscopic damage scoring system previously described. In brief, criteria include submucosal edema, epithelial hyperplasia, epithelial integrity, neutrophil, and mononuclear cell infiltration (27, 28). A 5-point scale was given on each item as follows: 0, no sign of inflammation; 1, mild damage; 2, moderate damage; 3, severe damage; and 4, maximal damage.

### Immunofluorescence Analysis

Serial sections (4 μm in thickness) mounted on probe-on slides were deparaffinized in xylene and rehydrated in a series of graded ethanol solutions. The sections were then rinsed in PBS and washed with 0.3% Triton X-100 for 20 min, followed by incubation in PBS containing 0.5% bovine serum albumin (BSA) for 0.5 h at room temperature. This blocking step was followed by incubation with appropriate dilutions of primary antibodies against E-cadherin (1:50, ProteinTech, Chicago, IL, USA), β-catenin (1:50, Cell Signaling, Danvers, MA, USA), and ZO-1 (1:200, Thermo Fisher Scientific, Waltham, MA, USA) overnight at 4°C. Sections were then washed 3 times for 5 min at room temperature followed by incubation with the fluorochrome-conjugated secondary antibodies for 3 h at room temperature. After being incubated for 7 min with 1:1,000 Hoechst33258 in PBS, the tissue was washed 3 times for 5 min with PBS at room temperature and mounted with glycerol and glass coverslips. Preparations were stored at −20°C until images were acquired using the BX43 Upright Microscope (Olympus, Tokyo, Japan) with the DP74 camera (Olympus) or Zeiss LSM 880.

### Western Blotting Analysis

Total protein of the intestinal tissues was extracted from each group using ice-cold radioimmunoprecipitation assay (RIPA) buffer (Beyotime Biotechnology, Shanghai, China), with added protease and phosphatase inhibitors (Thermo Fisher Scientific, USA). After ultracentrifugation at 120,000×*g* at 4°C for 30 min, the supernatant proteins were collected and stored at a −80°C refrigerator. After being boiled for 10 min, the protein was electrophoresed on 8% polyacrylamide gel and transferred to polyvinylidene fluoride membranes (Millipore, Darmstadt, Germany). After incubation with 5% non-fat milk in TBST (TBS with 0.1% Tween-20) for 2 h at room temperature, the membranes were incubated with primary antibodies (ZO-1, 1:500, Thermo Fisher; E-cadherin, 1:1,000, ProteinTech; and β-catenin, 1:1,000, Cell Signaling) overnight at 4°C. The membranes were washed in TBST several times, then incubated with horseradish peroxidase-conjugated secondary antibodies for 2 h at room temperature, and visualized with enhanced chemiluminescence (Thermo Fisher Scientific, USA). Proteins were normalized to GAPDH.

### Statistical Analysis

GraphPad Prism 9.0.0 software (GraphPad Software Inc., San Diego, CA, USA) was carried out to perform the statistical analyses. Age was described as mean ± SDs and compared by unpaired t-test, for the statistics that passed the normality test (D’Agostino–Pearson normality test). Numbers and percentages were used to present the frequency of gender and the difference between groups compared by Fisher’s exact test. Continuous variables were described as mean and/or median and interquartile range, depending on the outcome of a D’Agootino–Pearson normality test, and compared by Student’s t-test or the Mann–Whitney test, respectively. Receiver operating characteristic (ROC) analysis was performed to determine the cutoff values of the score of GSRS. Spearman’s correlation analysis was used to determine the correlations between the levels of the different analytes. A *p*-value < 0.05 was considered statistically significant.

## Results

### Gut Dysfunction and Gut Inflammation in Cryptogenic Stroke Patients

**Figure 1:**
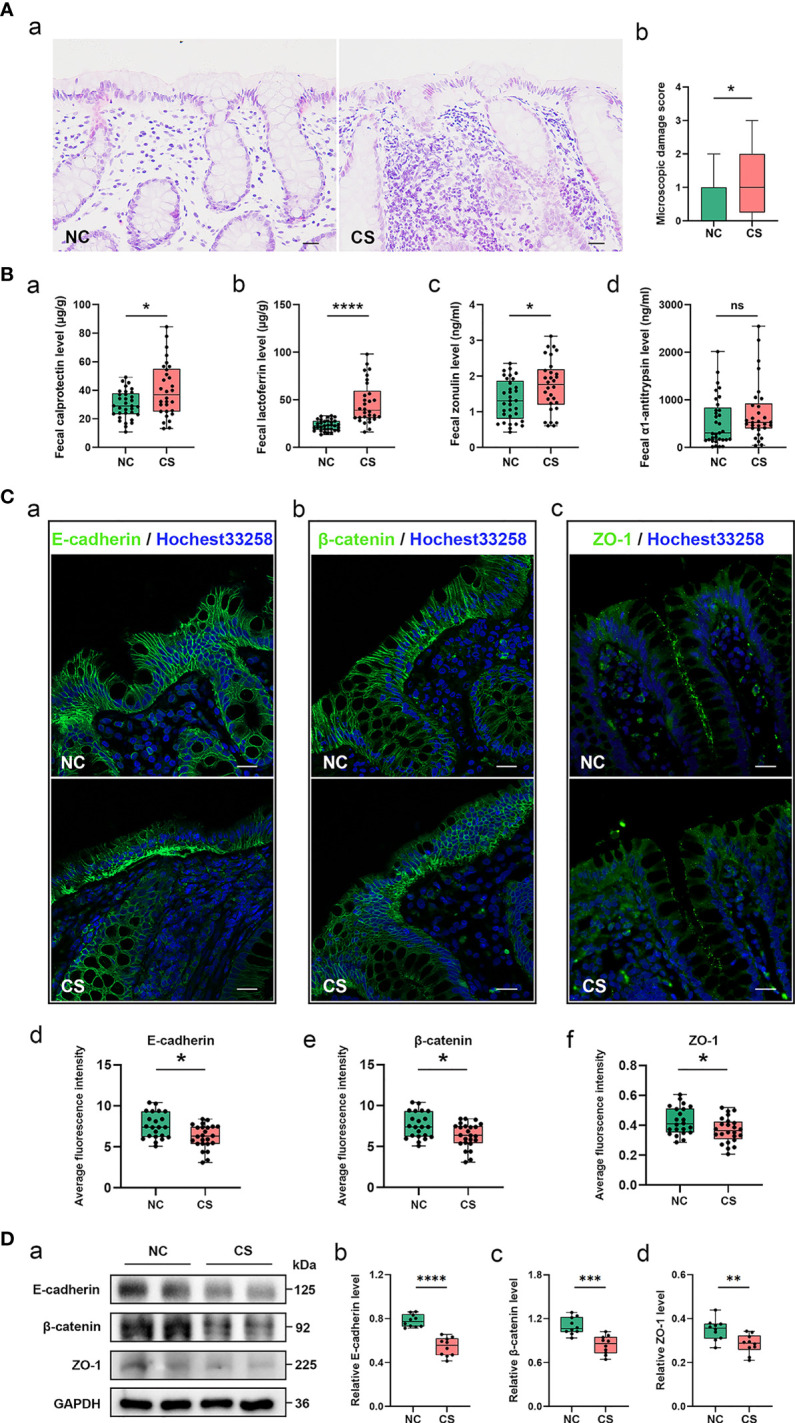
Gut inflammation with increased intestinal permeability developed in CS patients. **(A)** Representative images of H&E staining of the colon tissue **(a)**. Microscopic damage score calculated after microscopic analyses of the colon sample from two groups **(b)**. Scale bar: 20 μm. **(B)** Comparison of fecal biomarker levels [calprotectin **(a)**, lactoferrin **(b)**, zonulin **(c)**, and α1-antitrypsin **(d)**] in CS patients and the controls. **(C)** Representative image and quantification of E-cadherin **(a, d)**, β-catenin **(b, e)**, and ZO-1 **(c, f)** in immunofluorescence staining. Scale bar: 20 μm. **(D)** Representative immunoblot images of E-cadherin, β-catenin, and ZO-1 in the colonic epithelium of subject **(a)** and quantifications **(b–d)**. The loading controls (GAPDH) were run on different gels in the same experiment. n = 10; *p* < 0.05 indicates statistical significance (*p < 0.05, **p < 0.01, ****p < 0.0001), and ns means *p* ≥ 0.05. Results are expressed as median and quartile. CS, cryptogenic stroke.

The patients’ demographic and clinical information in the two groups were summarized in **Table 1**. In the study, GSRS was used to assess the severity of GI dysfunction in CS patients, which is a validated questionnaire based on the occurrence and intensity of GI symptoms experienced during the past week. We found that total GSRS scores were significantly higher in the CS group compared with NCs (17.33 vs. 12.70, *p* = 0.0028). GI dysfunction was found in 40% of CS patients, with the cutoff value of GSRS score of 19.50. H&E staining of the colonic mucosa showed the inflammatory-related morphological changes (including the accumulation of leucocytes and damaged intestinal epitheliums) that occurred in the colon mucosa in 16/24 CS patients but none of the NCs, despite that cryptitis was not found in all of the subjects (**Figure 1A**).

**Table 1:** Characteristics of the study participants.

	NCs (n = 33)	CS patients (n = 30)	*p*-Value
Mean age (years ± SD)	41.33 ± 8.07	40.93 ± 8.57	0.8494
Male sex (n, %)	20, 60.61	17, 56.67	0.8017
BMI (mean, SD)	24.34, 2.31	24.55, 2.02	0.7820
Hypertension (n, %)	6, 18.18	9, 30.00	0.3763
Hyperlipidemia (n, %)	5, 15.15	8, 26.67	0.3627
Diabetes mellitus (n, %)	3, 9.10	5, 20.00	0.4616
Smoking status			
Present (n, %)	9, 30.00	11, 36.67	0.5921
Past (n, %)	12, 36.36	14, 46.67	0.6073
CRP (mean, SD)	0.80, 0.38	1.64, 0.87	<0.0001
WBC count (mean, SD)	6117.58, 859.92	6828.67, 1350.89	0.0498
GSRS (mean, SD)	12.70, 4.90	17.33, 6.39	0.0028
NIHSS (mean, SD)	–	12.37, 5.93	–
Infarct volume (mean, SD; n = 23)	–	7.39, 3.45	–

BMI, body mass index; CRP, C-reactive protein; WBC count, white blood cell count; GSRS, Gastrointestinal Symptom Rating Scale; NIHSS, the National Institutes of Health Stroke Scale; NCs, normal controls; CS, cryptogenic stroke.

Meanwhile, two fecal markers of intestinal inflammation (fecal calprotectin (FC) and fecal lactoferrin (FL)) and two fecal biomarkers of intestinal permeability (fecal zonulin (FZ) and fecal alpha-1-antitrypsin (Fα1-AT)) in fecal samples of all participants were tested (**Figures 1Ba**–**d**). Compared to NCs, the fecal samples showed significantly elevated FC concentrations (µg/g) in CS patients: 40.31 (13.22–84.51) vs. 29.97 (10.86–49.21), *p* < 0.05. FL levels (μg/g) were also significantly higher in CS patients: 46.53 (16.32–98.02) vs. 23.32 (13.53–33.30), *p* < 0.0001. FZ levels (ng/ml) were found a significant elevation in CS patients compared with NCs: 1.74 (0.61–3.12) vs. 1.33 (0.44–2.36), *p* < 0.05. The level of Fα1-AT, albeit not a statistically significant difference, shows a trend towards elevated concentrations in CS patients compared to NCs: 730.32 (41.56–2,549.84) vs. 546.74 (16.81–2,015.63), *p* > 0.05.

The integrity of the intestinal epithelial barrier (IEB) was also investigated, which serves as the first boundary of defense between blood circulation and the luminal environment. Thus, the expression of E-cadherin, β-catenin, and ZO-1, major components of adherens junctions (AJs) and tight junctions (TJs), were investigated in colonic epithelium by fluorescence analysis (**Figure 1C**). Colonic samples from 22 NCs and 24 CS subjects were finally analyzed because samples from 3 of the controls and 2 of the patients were excluded, as the mucosa was too small or too damaged to allow a reliable analysis. A normal expression of E-cadherin was observed in the colonic samples of all of NCs and only 6/24 CS patients, while a loss of E-cadherin was found in the mucosa of 18 out of 24 CS patients. Similar results were observed in β-catenin staining. Moreover, ZO-1 morphology was disrupted in the mucosa of 3 out of 22 controls and 17/24 CS patients. Downregulated expressions of E-cadherin, β-catenin, and ZO-1 were also observed by Western blotting analysis (**Figure 1D**), which further confirmed the increased permeability of colonic epithelium in CS patients.

### Enhanced Systemic Inflammatory Response Is Associated With Gut Inflammation in Cryptogenic Stroke Patients

**Figure 2:**
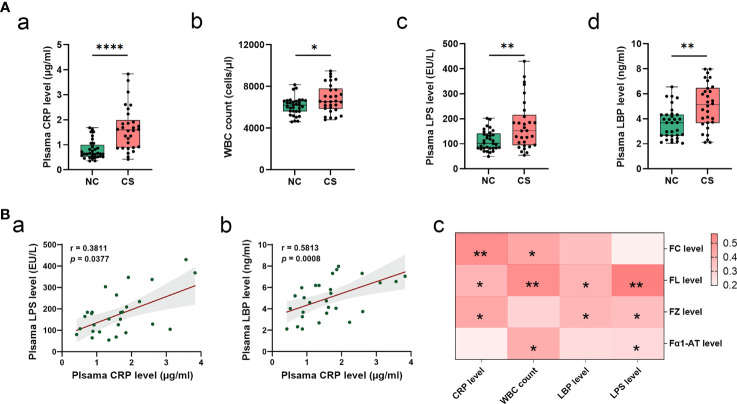
Systemic inflammatory in CS patients. **(A)** Comparison of systemic inflammatory biomarker levels (CRP, WBC count, LPS, and LBP) in CS patients and the controls. Results are expressed as median and quartile. **(B)** Spearman’s correlation analysis between plasma LPS and LBP levels and plasma CRP levels **(a, b)**. Heatmap of Spearman’s correlation analysis between systemic inflammatory indices (LPS, LBP, CRP, and WBC count) and fecal inflammatory biomarkers (FC, FL, and FZ, and Fα1-AT) **(c)**. *p* < 0.05 indicates statistical significance (*p < 0.05, **p < 0.01, ****p < 0.0001). CS, cryptogenic stroke; CRP, C-reactive protein; WBC, white blood cells; LPS, lipopolysaccharide; LBP, lipopolysaccharide-binding protein; FC, fecal calprotectin; FL, fecal lactoferrin; FZ, fecal zonulin; Fα1-AT, fecal alpha-1-antitrypsin.

From the analysis of the clinical data, it was found that the subjects from the CS group had higher levels of plasma CRP and WBC counts (**Table 1** and **Figures 2Aa, b**). This indicated that there was a systemic inflammatory response after CS. Given that high-frequency GI dysfunction and gut inflammation with increased permeability happened in CS patients, we then test the plasma level of bacterial LPS, another direct biomarker of systemic inflammation that is secreted from Gram-negative bacteria. Plasma LBP was also quantified to allow a reliable analysis, as the LBP measurements are not subject to contamination. Plasma levels of bacterial LPS and LBP were not only higher in CS patients compared to the controls (**Figures 2Ac, d**) but also positively related to each other. Plasma LPS and LBP levels were both positively correlated with CRP levels (r = 0.3811, *p* = 0.0377, and r = 0.5813, *p* = 0.0008, respectively) (**Figures 2Ba, b**). Meanwhile, the levels of plasma CRP, LPS, and LBP levels and WBC counts were found to have a significantly positive correlation with the levels of fecal markers, including FC, FL, FZ, and Fα1- AT (**Figure 2Bc**).

### Differential Gut Microbiota Between Cryptogenic Stroke Patients and Normal Controls

**Figure 3:**
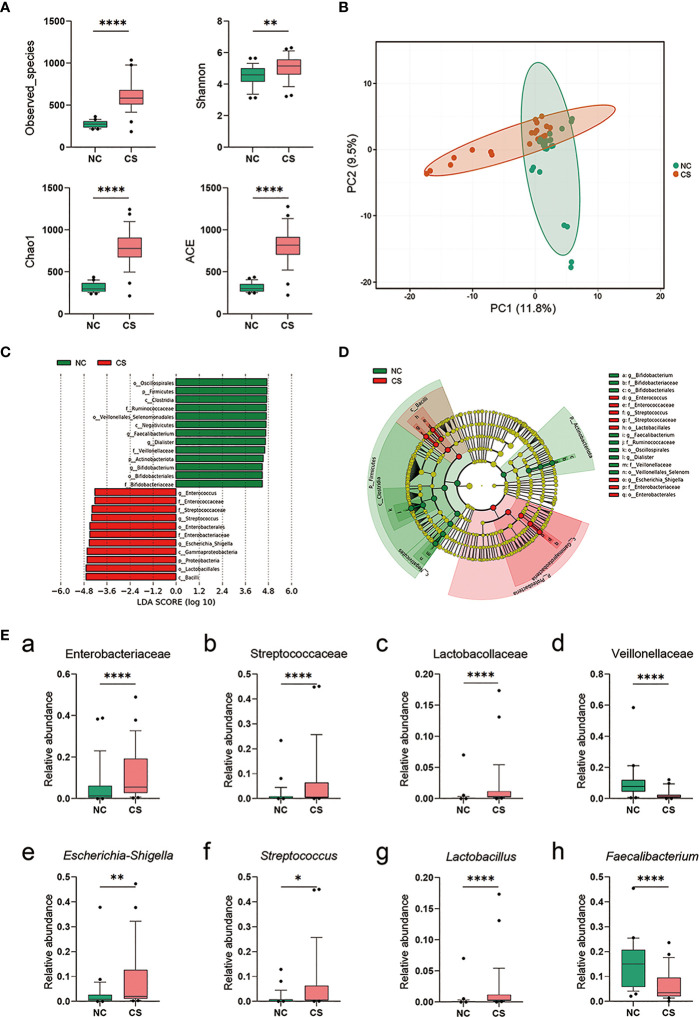
Comparison of the representative taxonomic abundance between CS patients and NCs. **(A)** Alpha diversity of gut microbiota between CS patients and NCs. The Observed_species, Chao1, Shannon, and ACE index values. **(B)** PCA scores based on the relative abundances of OTUs (at the 97% similarity level). **(C)** Histogram of the linear discriminant analysis (LDA) scores for differentially abundant bacterial taxa between two groups (LDA score threshold: ≥4.0). **(D)** Cladogram of the LEfSe analysis of the gut microbiota in NC group (green) and CS group (red). Rings from the inside out represent taxonomic levels from phylum to genus, and sizes of the circles indicate the relative abundance levels of the taxa. **(E)** Relative abundance of intestinal microbiota at the family level **(a–d)** and genus level **(g–h)**. *p* < 0.05 indicates statistical significance (*p < 0.05, **p < 0.01, ****p < 0.0001). CS, cryptogenic stroke; NCs, normal controls; PCA, principal component analysis; OTUs, operational taxonomic units; LEfSe, linear discriminant analysis effect size.

To investigate if the gut microbiota altered in CS patients, we compared the composition and diversity of fecal microbiomes between CS patients and NCs by pyrosequencing the bacterial 16S ribosomal RNA gene. According to strict inclusion and exclusion criteria, 27 CS patients and 27 NCs were enrolled and completed the analysis. The alpha diversity indices, including Observed_species, Chao1, Shannon, and ACE, were found to be significantly higher in CS patients than NCs (**Figure 3A**), while there was no difference in the Simpson index. These results suggest that the richness and diversity of the gut microbiotas were significantly higher in CS patients. Principal component analysis (PCA) also showed a significant difference between the two groups (**Figure 3B**). Linear discriminant analysis effect size (LEfSe) analysis was performed to identify the differences of abundant bacterial taxa between two groups (**Figures 3C, D**). At the family level, a significantly higher abundance of Enterobacteriaceae, Streptococcaceae, and Lactobacillaceae and a lower abundance of Veillonellaceae were observed in the CS group compared to the NCs (**Figures 3Ea**–**d**). At the genus level (**Figures 3Ee**–**h**), there was an increased abundance of *Escherichia–Shigella*, *Streptococcus*, *Lactobacillus*, and *Klebsiella* in the CS group with a decrease in *Faecalibacterium*, *Dialister*, and *Roseburia*.

### Gut Dysbiosis Was Related to Systemic Inflammation and the Stroke Severity and the Infarct Volumes

**Figure 4:**
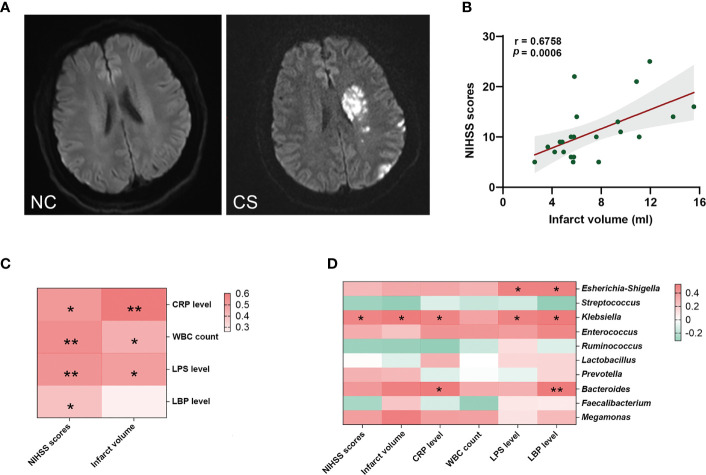
Gut microbiota is associated with systemic inflammatory response levels and the severity of stroke. **(A)** Representative DWI image of the NCs (left) and CS patients (right). **(B)** The NIHSS scores positively related to the infarct volumes. **(C)** Heatmap of Spearman’s correlation analysis between systemic inflammatory factors and the severity of stroke. **(D)** Heatmap of Spearman’s correlation analysis between the abundance of gut microbiota and systemic inflammatory factors and the severity of stroke. *p* < 0.05 indicates statistical significance (*p < 0.05, **p < 0.01). DWI, diffusion-weighted imaging; CS, cryptogenic stroke; NCs, normal controls; NIHSS, National Institutes of Health Stroke Scale.

The stroke severity of patients was measured by NIHSS at admission, and the mean NIHSS score of patients was 12.37 (SD: ± 5.93). Another marker of stroke severity was shown as the size of infarct volumes, which was measured on DWI (**Figure 4A**) and was available in 23 CS patients (76.6%) in this study. The mean infarct volumes of the patients were 7.39 ml (SD: ± 3.45). We found a positive correlation between NIHSS scores and infarct volumes in CS patients (r = 0.6758, *p* = 0.0006) (**Figure 4B**). We also found that patients with a more severe admission NIHSS and larger infarct volumes had higher systemic inflammatory markers (including CRP, WBC, LPS, and LBP) (**Figure 4C**). Spearman’s correlation analysis was carried out to evaluate the potential relationship between the gut microbiome and systemic inflammatory markers and the severity of stroke (**Figure 4D**). The results indicated that as the abundance of *Klebsiella*, *Escherichia–Shigella*, and *Bacteroides* of the patients increased, so did the plasma levels of their systemic inflammatory markers. Meanwhile, the abundance of *Klebsiella* was significantly positive correlated with NIHSS scores (r = 0.3853, *p* = 0.0471) and infarct volumes (r = 0.5079, *p* = 0.0222). However, no significant correlation was found between *Escherichia–Shigella* or *Bacteroides* and stroke severity (neither NIHSS scores nor infarct volumes). These results suggest that the expansion of *Klebsiella* is correlated to the severity of stroke and the enhanced the inflammatory response.

## Discussion

In this study, we found that the gut microbiome of CS patients was disordered as compared to NCs. Moreover, CS patients had increased systemic inflammatory response and imbalanced gut homeostasis. Importantly, we reveal that higher abundance of *Klebsiella* is positively correlated to systemic inflammation and stroke severity of CS patients. These results suggest that gut microbiota is associated with the severity of CS and the gut and systemic inflammatory response.

The high prevalence of GI symptoms in patients with ischemic stroke has been reported in several studies (9, 10). By comparing the scores of GSRS, we found more severe GI dysfunction in CS patients compared to the NCs. To seek the cause of GI dysfunction, histological staining of the colon mucosa and fecal biomarkers testing was performed in the study. We found gut inflammation in CS patients with inflammatory cells accumulated in the colon mucosa and the increasing levels of fecal biomarkers. Fecal biomarkers are a useful non-invasive way of identifying intestinal inflammation, including FC and FL (29–31). Both FC and FL could be reliably measured because they can remain stable in stool by their character (30). Calprotectin, a protein evenly distributed through the feces, is in proportion to the degree of inflammation (32) and resistant to intestinal bacterial degradation (33). Lactoferrin is a primary factor in the acute inflammatory response (34, 35). FL levels quickly increase with the influx of neutrophils during intestinal inflammation and have antibacterial activity. These results suggested that gut inflammation occurred in CS patients.

Several studies have attributed intestinal inflammation to a loss of AJs and TJs (36–38). As the major component of IEB, AJ and TJ proteins with integrality and normal distribution control the passage of various substances through the intestinal epithelium (39). E-cadherin and β-catenin are both important components for the maintenance of AJs (37), and ZO-1 presents the key component of tight junction (40). In the present study, we found that both AJs and TJs including E-cadherin, β-catenin, and ZO-1 were downregulated in CS patients, which indicated that increased intestinal permeability with IEB damage happened in CS patients. The finding is in line with findings from stroke mouse models that reported the gut inflammation with downregulation and broken TJs of the intestinal mucosa (41) and negative changes in intestinal structure and function that happened after stroke (42).

By analyzing the clinical data, we found a significantly higher level of plasma CRP and WBC count in CS patients, which suggests that there might be a systemic inflammatory response in CS patients. Higher plasma LPS and LBP levels supported this finding because they were reported associated with systemic inflammation (43, 44). Similar results have been reported in other studies (45, 46); however, the subjects included other types of strokes except the cryptogenic. Therefore, we provide evidence that systemic inflammation happened after brain infarction in CS patients. LPS, the endotoxin portion of the Gram-negative bacterial outer membrane, has a short half-life of just a few hours in plasma. Thus, the finding of increased plasma LPS levels suggests a systemic inflammatory response with a continuous release of LPS into the blood in CS patients. Taking into account that the loss of IEB integrity could promote LPS translocation from the intestinal lumen into the circulatory system (47), we speculate that the source of LPS in plasma might be the gut. The enhanced levels of plasma LBP also support this. LBP has been reported to correlate with colonic permeability, and the enhancing plasma LBP levels possibly reflect the impact of systemic inflammation from gut leakiness (44). To confirm our speculation, we performed the analysis of gut microbiota and found a disturbance in the gut microbiome with an increasing level of several Gram-negative bacteria in this study, especially in the Enterobacteriaceae family with increasing abundances of *Escherichia–Shigella* and *Klebsiella*. *Escherichia–Shigella* has high pathogenicity and infectivity and can produce strong endotoxins, increase intestinal permeability, exacerbate colitis, and cause endotoxemia. *Klebsiella* is one of the most important pathogenic bacteria that cause pneumonia, respiratory infections, peritonitis, diarrhea, and septicemia. Findings from a study are similar to ours and indicated that the resource of LPS in stroke patients might be gut bacteria, with the LPS identified being from *Escherichia coli* O111:B4 (46). Therefore, the systemic inflammatory response is associated with gut dysbiosis and gut inflammation with increased intestinal permeability.

Animal studies indicated that intestinal microbiota disturbance plays a vital role in the severity of ischemic stroke (48). Studies have found that depletion of gut microbiota *via* antibiotic administration decreased the survival rate of stroke in a mouse model (49). Moreover, in the middle cerebral artery occlusion model, Benakis et al. found that gut dysbiosis would affect the outcome of ischemic stroke (50). Though dysregulation of the microbiota has been identified (51–53), few studies paid attention to the effect of gut dysbiosis on the severity of stroke in patients, let alone CS patients. In this study, we found the genus *Escherichia–Shigella* and *Klebsiella* were significantly positively correlated with NIHSS scores and infarct volumes in CS patients, respectively. No significant correlation was found between *Streptococcus* abundance and NIHSS scores or infarct volumes, although it is shown that with *Streptococcus* increased, the NIHSS scores or infarct volumes were also increased. Overall, our results suggest that symptom severity of CS is associated with gut microbiota disturbance and enhancing gut and systemic inflammatory reaction.

From the above findings, we gain a more comprehensive understanding of the role of the gut microbiota in the pathological process of CS. There is a wide link between gut dysbiosis and central nervous system (CNS) diseases; thus, many therapies of adjusting gut microbiota have been developed, including manipulation of the diet, ingestion of prebiotics and probiotics, and fecal microbiota transplantation, to modulate the gut microbiota and associated metabolites. In the future, we will try to adjust the microbial compositions to confirm whether it could improve the prognosis of CS.

Growing amounts of evidence support that the alterations of gut microbiota have been linked to the pathology of CNS diseases (54–56). Recent evidence suggests that cross-talk between the gut microbiome and the immune system is important for gut–brain communication in neuropsychiatric and neurodegenerative disorders (57) and systemic inflammation may have a contribution to the outcome or progression of neurodegenerative disease (58, 59). However, few studies focus on the communication between gut microbiota and systemic inflammation and their roles in cerebrovascular diseases. In the present study, we found their role in CS patients by exploring the correlation between gut microbiota, systemic inflammatory factors, and the severity of stroke.

However, there were certain limitations in this study. First, the participants of this study were from a single center with a small sample size. Large-sample and multicenter studies are still needed to confirm the results in the future. Another drawback is that our research was not involved in the effect of gut microbiota on the prognosis of CS because no further follow-up was performed. Third, although the samples were collected before treatment of stroke, the gut microbiome could have been influenced by other confounders such as diet, exercise, and risks of stroke (such as hypertension and diabetes) (60, 61). Although this study has some limitations, it is a beneficial exploration of CS, which may provide a foundation for subsequent studies.

## Data Availability Statement

The datasets presented in this study can be found in online repositories. The names of the repository/repositories and accession number(s) can be found below: SRA, PRJNA790465.

## Ethics Statement

The studies involving human participants were reviewed and approved by the Institutional Ethics Committees of The First Affiliated Hospital of Zhengzhou University. The patients/participants provided their written informed consent to participate in this study.

## Author Contributions

XD conceived and designed the experiments. XD coordinated the whole project. XD, QZ, and JT and XW were responsible for the initial assessment and diagnosis of patients. QZ, YC, YZ, and LM were responsible for assessing, documenting their patients' health information. QZ, YC, HL, and KS collected the samples of participants. HT, RF, JW, RZ and LG performed the image analysis. QZ, EW, LM, HT and YW performed statistical analysis. XD, EW, QZ, YC, YZ, and LM participated in the final data analysis and interpretation. XD, QZ, and YC did most of the writing with input from other authors. All authors discussed the results and commented on the manuscript.

## Funding

XD was supported by grants from the National Natural Science Foundation of China (no. 82171248).

## Conflict of Interest

The authors declare that the research was conducted in the absence of any commercial or financial relationships that could be construed as a potential conflict of interest.

## Publisher’s Note

All claims expressed in this article are solely those of the authors and do not necessarily represent those of their affiliated organizations, or those of the publisher, the editors and the reviewers. Any product that may be evaluated in this article, or claim that may be made by its manufacturer, is not guaranteed or endorsed by the publisher.
